# Semaglutide in Heart Failure: A Systematic Review of Outcomes of Semaglutide in Heart Failure Patients

**DOI:** 10.7759/cureus.64668

**Published:** 2024-07-16

**Authors:** Nishtha Gupta, Tesingin D Uwawah, Kamaldeep Singh, Hritik Madan, Siddharth Kumar, Bharat Midha, Kriti Soni, Aparjit Singh, Amandeep Bhogal, Arpit Jain

**Affiliations:** 1 Internal Medicine, Kasturba Medical College, Mangalore, Manipal Academy of Higher Education, Mangalore, IND; 2 Internal Medicine, Cherubin Family Health Care, Brooklyn, USA; 3 Internal Medicine, Jawaharlal Nehru Medical College, Belgaum, IND; 4 Internal Medicine, Adesh Medical College and Hospital, Ambala, IND; 5 Internal Medicine, Armed Forces Medical College, Pune, IND; 6 Internal Medicine, Government Medical College & Hospital, Chandigarh, IND; 7 Internal Medicine, Subharti Medical College & Hospital, Meerut, IND

**Keywords:** glp-1 agonist, obesity, diabetes, heart failure, semaglutide

## Abstract

Heart failure (HF) stands as a formidable challenge in global healthcare casting a long shadow over both morbidity and mortality. A significant interplay between HF and type 2 diabetes (T2DM) manifests an elevated risk of adverse cardiovascular events in T2DM patients. Glucagon-like peptide 1 emerges as a pivotal player in T2DM, which is released in response to meals rich in glucose and lipids. We aim to assess the outcomes of using semaglutide in HF. A comprehensive literature search was performed using electronic databases, including PubMed/Medline, the Cochrane Library, and Google Scholar, covering all records up to May 10, 2024. Studies meeting inclusion criteria were selected. Qualitative analysis was conducted to analyze the findings of the studies included. Four studies (three randomized controlled trials and one observational study) were included in our manuscript. There was a significant decrease in the Kansas City Cardiomyopathy Questionnaire clinical summary score (p< 0.001), body weight (p< 0.001), six-minute walk distance (p< 0.001), and CRP levels (p< 0.001). A statistically significant decrease in overall major adverse cardiac events was observed with a hazard ratio of 0.76 (95% CI 0.62, 0.92). Other factors and adverse effects were also discussed in our manuscript. Our study showed that semaglutide resulted in improvement in HF patients. Although adverse effects were observed, they were not as significant as the placebo itself.

## Introduction and background

Cardiovascular diseases stand as a formidable challenge in global healthcare casting a long shadow over both morbidity and mortality [1]. According to a 2020 report by the American Heart Association, an alarming 6.2 million American citizens grappled with this pervasive condition, underscoring its status as a critical public health concern [1,2]. The literature has proved a significant interplay between cardiovascular diseases and type 2 diabetes mellitus (T2DM) manifesting an elevated risk of major adverse cardiovascular events (MACEs) in T2DM patients. A staggering 65% of deaths in T2DM patients are directly attributed to cardiovascular diseases, adding urgency to the exploration of innovative therapeutic interventions [3,4].

Management of T2DM has witnessed significant evolution over the years. While lifestyle modifications remain a mainstay in T2DM management, the arsenal of pharmacological interventions has expanded. Current treatment for the management of type 2 diabetes includes lifestyle changes and medications including metformin, sulfonylureas, glinides, thiazolidinediones, GLP-1 agonists, DPP-4 inhibitors, SGLT-2 inhibitors, and insulin analogs [5]. Still, the quest for optimal glycemic control is contaminated with the challenge of managing cardiovascular complications, which contribute substantially to the morbidity and mortality associated with T2DM [3]. Sulfonylureas and DPP-4 inhibitors are commonly prescribed anti-diabetic medications and have been assessed for their impact on cardiovascular outcomes. A study assessing sulfonylureas and DPP-4 inhibitors' effect on cardiovascular outcomes showed no significant (hazard ratio (HR) 0.95 [0.80-1.14]; 95% CI; p= 0.604) difference in overall MACEs (including myocardial infarction, stroke, unstable angina, and hospitalization due to other cardiovascular causes) in patients with type 2 diabetes [6]. On the other hand, another study showed that insulin treatment was a significant (HR 2.10 [1.42-3.10]; 95% CI; p= 0.0002) risk factor for MACEs, conveying the complexity in managing T2DM and highlighting the need for alternative therapeutic approaches [7].

Recently, glucagon-like peptide 1 (GLP-1) agonists, including semaglutide and liraglutide, have attracted attention for their potential effects on both glycemic control and cardiovascular health and have shown favorable effects in the reduction of MACE in type 2 diabetic patients [8]. The observation of GLP-1 agonist’s impact on cardiovascular outcomes is particularly significant given the diverse galaxy of existing anti-diabetic medications and the need to identify therapies that not only manage glycemic control but also manage the increased T2DM-associated cardiovascular risks. GLP-1 emerges as a pivotal player in T2DM, which is released in response to meals rich in glucose and lipids. GLP-1 orchestrates the regulation of postprandial insulin secretion and glucose concentrations in the plasma [9]. It was unveiled two decades ago that the concentration of GLP-1 remains notably preserved in individuals grappling with T2DM [10]. This revelation paved the way for the development and scrutiny of drugs linked to this hormone, constituting a promising frontier in the management of T2DM [11].

Among the cohort of GLP-1 receptor agonists, semaglutide is a distinguished drug [12]. Semaglutide is the first GLP-1 agonist to gain approval from the FDA in both oral and subcutaneous formulations to be used in the management of T2DM [12]. The oral formulation of semaglutide shows somewhat similar effects on glycemic control and reducing body weight in trials as its subcutaneous counterpart [12]. Originally designed for the management of the said disease, semaglutide, akin to its analogs, has transcended its primary purpose, venturing into the domain of heart failure (HF) management [13].

The precise mechanism by which semaglutide mitigates adverse cardiovascular effects remains shrouded in complexity, prompting an array of proposed pathways [14]. The literature is advancing to unravel this intricate mechanism of semaglutide in managing cardiovascular diseases including non-fatal myocardial infarction and non-fatal stroke and to understand and comprehensively evaluate the efficacy, safety, and therapeutic outcomes of semaglutide in the management of MACE among those with T2DM and cardiovascular diseases [15-17].

In this comprehensive systematic review, we evaluate existing knowledge and recent advancements, to provide a comprehensive understanding of semaglutide's role in addressing the critical relationship of T2DM and cardiovascular complications. Through an in-depth analysis of clinical studies, mechanisms, and their therapeutic outcomes, we aim to gather valuable insights and provide a comprehensive overview of semaglutide's impact on the cardiovascular health in T2DM patients that can inform clinical practice and guide future research in the pursuit of effective management strategies for individuals facing the burden of T2DM and adverse cardiovascular outcomes.

## Review

Materials and methods

Search Strategy and Databases

The systematic review adhered to the Preferred Reporting Items for Systematic Reviews and Meta-Analyses (PRISMA) guidelines [18]. Electronic searches were conducted in PubMed/Medline, the Cochrane Trial Register, and Google Scholar from their inception until May 10, 2024. The search string utilized was: (Semaglutide) AND (heart failure OR HF). The first available literature reviewing semaglutide in HF dates back to 2016, due to the novelty of such clinical application. Additionally, we examined the references of previously published meta-analyses, cohort studies, and review articles to identify pertinent studies.

Study Selection Criteria

Studies were selected to be in accordance with our PICOS; P (Patients): Patients who were either at risk of or already had experienced an episode of HF; I (Intervention): Semaglutide; C (Control): Placebo; O (Outcomes): To assess the efficacy of semaglutide in HF patients; S (Studies): Observational studies and randomized controlled trials (RCTs).

Studies were excluded if they were non-humanized studies (studies not conducted in human participants), case reports, case series, review articles, protocols, predesigned analyses or the outcomes were not suitable for our study.

Data Extraction and Quality Assessment 

Two reviewers screened the electronic databases, and the studies were imported into EndNote Reference Library version 20.0.1 (Clarivate Analytics, London, UK) for duplicate removal. Data from the selected studies were extracted by two investigators and entered into a computer spreadsheet.

The quality and bias of the studies were assessed using the Newcastle-Ottawa Scale (NOS) for observational studies and the Cochrane Collaboration Tool for clinical trials. An NOS score of 1-5 indicated a high risk of bias, 6-7 indicated a moderate risk, and a score above 7 indicated a low risk of bias.

The Cochrane Collaboration's tool evaluated seven domains: sequence generation, allocation concealment, blinding of participants and personnel, blinding of outcome assessment, completeness of outcome data, selective outcome reporting, and other potential biases. Each domain, along with the overall risk of bias, was classified as high, unclear, or low. Based on these assessments, the overall quality of evidence was categorized as high, moderate, or low risk of bias.

Statistical Analysis

The narrative approach was used to analyze the findings of the articles due to heterogeneity between them in their outcome presentation.

Results

Search Strategy

**Figure 1:**
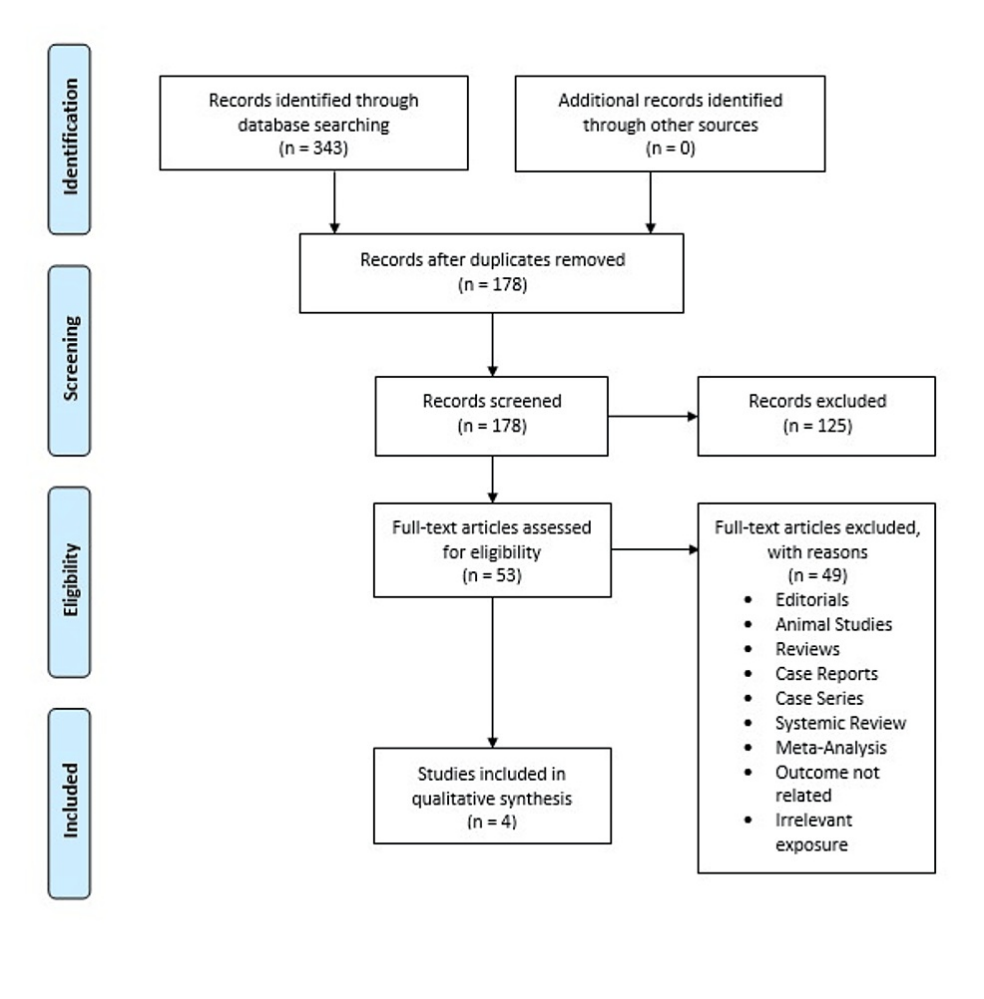
PRISMA flow diagram illustrating the summary of literature search. PRISMA: Preferred Reporting Items for Systematic Reviews and Meta-Analyses

Three hundred and forty-three articles were retrieved from the three databases. After removing duplicates, 178 articles remained. The full text of 53 articles was read. Four articles were selected for our systematic review. Figure 1 shows the PRISMA flow chart.

Baseline Characteristics

Table 1 describes the baseline characteristics of the selected studies [19-22]. Four studies were included in our articles (three RCTs and one observational study). Eighteen thousand two hundred and ninety-six T2DM patients were included in our study out of which 10,904 patients took semaglutide. These studies in general had around the same age and gender in their cohort as seen in Table 1. Two studies were from the USA while one each was from Spain and Canada. The mean age was 65.07 years with 41.7% females in the studies. Two studies used only the subcutaneous route [19,21] while the other two studies used both oral and subcutaneous routes [20,22]. Details of the duration of studies, dosage, and duration of dosage are provided in Table 1.

**Table 1:** The baseline characteristics of the included studies

Study	Year	Study Design	Place of Study	Duration of Study	Total Population (N)	Patients Taking Semaglutide N (%)	Mean Age (Years)	Females N (%)	Route	Dose	Duration of Dose	Net Risk of Bias
Hussain et al., 2020 [20].	2020	Randomized clinical trial	Canada	2.1 and 1.3 years	6,480	3,239	65.4	2,302 (35.5)	Oral and subcutaneous	Oral 14mg; Subcutaneous 0.5mg and 1.0mg	Oral- once-daily subcutaneous- Once-weekly	Low risk
Perez-Belmonte et al., 2022 [21].	2022	Observational study	Spain	12 months	136	136	72.6 ± 11.0	62 (45.5)	Subcutaneous	Subcutaneous 0.25 mg for 4 weeks, increased to 0.5mg for following 4 weeks until maintenance dose 0.5mg or 1.0mg	Once-weekly	Low risk
Kosiborod et al., 2023 [19].	2023	Randomized clinical trial	USA	52 weeks	529	263	-	-	Subcutaneous	Subcutaneous 2.4mg	Once-weekly	Low risk
Aroda et al., 2023 [22].	2023	Randomized clinical trial	USA	70 and 83 weeks	11,151	7,266	57.2	4,932 (44.2)	Oral and subcutaneous	Oral 3mg, 7mg, and 14mg; Subcutaneous 0.5mg and 1.0mg	Oral- once-daily Subcutaneous- once-weekly	Low risk

Quality Assessment 

**Figure 2:**
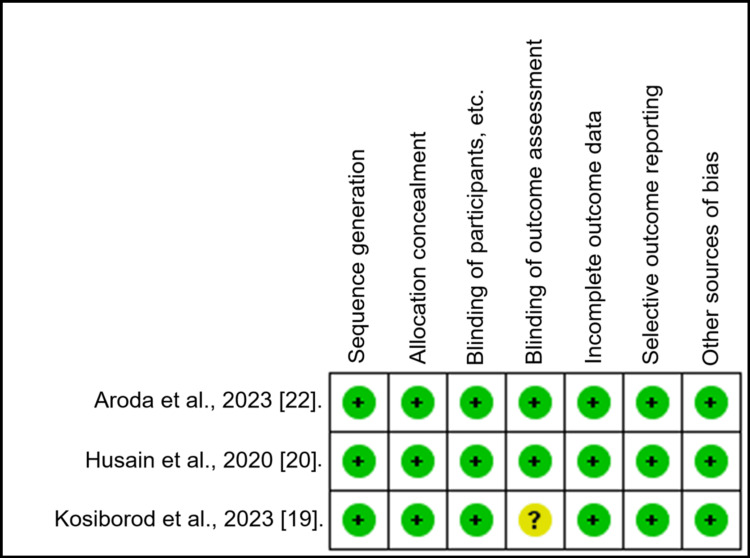
Risk of bias summary for the RCT +: low risk of bias; ?: unclear risk of bias; -: high risk of bias; RCT: randomized controlled trial

All studies have a low risk of bias (Tables 2, 3 and Figure 2).

**Table 2:** Demonstration of the quality assessment of the included cohort employing the Newcastle-Ottawa Scale

Study	Selection				Comparability	Outcome			Total Score
	Representation of exposed cohort	Selection of nonexposed cohort	Ascertainment of exposure	Outcome not present at the start of this study		Assessment of outcome	Length of follow-up	Adequacy of follow-up	
Perez-Belmonte et al., 2022 [21].	1	0	1	1	2	1	1	1	8

**Table 3:** Demonstration of the quality assessment of included RCTs employing the Cochrane Scale RCTs: Randomized controlled trials

	Sequence Generation	Allocation Concealment	Blinding of Participants, etc.	Blinding of Outcome Assessment	Incomplete Outcome Data	Selective Outcome Reporting	Other Sources of Bias	Net Risk
Husain et al., 2020 [20].	Low	Low	Low	Low	Low	Low	Low	Low
Aroda et al., 2023 [22].	Low	Low	Low	Low	Low	Low	Low	Low
Kosiborod et al., 2023 [19].	Low	Low	Low	Unclear	Low	Low	Low	Low

Efficacy of Semaglutide in HF

The efficacy of Semaglutide in HF patients was assessed using four articles [19-22].

Kosiborod et al. randomly assigned 529 patients with HFpEF and obesity to receive either once weekly semaglutide or placebo for a duration of 52 weeks [19]. The dual primary endpoints included the change from baseline in the Kansas City Cardiomyopathy Questionnaire clinical summary score (KCCQ-CSS) and the change in body weight. Confirmatory secondary endpoints comprised the change in the 6-minute walk distance, a hierarchical composite endpoint involving death and HF events, alterations in the KCCQ-CSS and 6-minute walk distance, as well as changes in C-reactive protein (CRP) levels. The results demonstrated a significant therapeutic effect of semaglutide in multiple key domains. The mean change in KCCQ-CSS was notably higher in the semaglutide group compared to placebo (16.6 vs. 8.7 points, respectively; estimated difference 7.8 points; 95% CI 4.8 to 10.9; P<0.001) [19]. Furthermore, semaglutide produced a remarkable mean percentage change in body weight of -13.3%, in contrast to -2.6% with placebo (estimated difference -10.7 percentage points; 95% CI -11.9 to -9.4; P<0.001) (Table 4) [19]. This signifies a substantial reduction in symptoms and physical limitations and a substantial weight loss associated with semaglutide therapy.

**Table 4:** Summary of clinical outcomes from studies on semaglutide in heart failure and diabetes management Dashes ("-") under "Comparator/Placebo Group": This indicates that either the comparator group was not applicable for that particular outcome measure or the specific data was not provided in the summarized information. Dashes ("-") under "Estimated Difference (95% CI) / Hazard Ratio (95% CI)": This signifies that either the study did not report an estimated difference or hazard ratio for that outcome measure, or it was not applicable (e.g., for categorical outcomes like adverse events). Dashes ("-") under "P-value": This indicates that either the study did not report a p-value for that specific outcome measure or it was not applicable (e.g., for safety outcomes or categorical data).

Study and Outcome Measure	Semaglutide Group	Comparator/Placebo Group	Estimated Difference (95% CI) / Hazard Ratio (95% CI)	P-value
Kosiborod et al. [19]				
Mean change in KCCQ-CSS	16.6 points	8.7 points	7.8 points (4.8 to 10.9)	<0.001
Mean percentage change in body weight	-13.30%	-2.60%	-10.7 percentage points (-11.9 to -9.4)	<0.001
Mean change in six-minute walk distance	21.5 m	1.2 m	20.3 m (8.6 to 32.1)	<0.001
Hierarchical composite endpoint win ratio	1.72	-	-	<0.001
Mean percentage change in CRP levels	-43.50%	-7.30%	-	<0.001
Serious adverse events	13.30%	26.70%	-	-
Pérez-Belmonte et al. [21]				
KCCQ total symptom score	79.9 points	59.0 points	-	<0.01
NYHA functional class III (baseline vs. 12 months)	16.2% vs. 40.4%	-	-	<0.01
NT-pro-BNP levels (pg/mL) (baseline vs. 12 months)	577.4 ± 322.1 vs. 969.5 ± 653.5	-	-	<0.01
HbA1c <7% (baseline vs. 12 months)	64.5% vs. 16.2%	-	-	<0.001
Body weight (kg) reduction	-12.7	-	-	-
BMI reduction (kg/m²)	-7.1	-	-	-
Adverse drug reactions	24.20%	-	-	-
Major adverse cardiovascular events (3P-MACEs)	6 events	-	-	-
Husain et al. [20]				
Incidence rate of MACE (events/100 subject-years)	3.1	4.2	0.76 (0.62, 0.92)	-
Fatal and non-fatal MI (hazard ratio)	-	-	0.89 (0.67, 1.18)	-
Fatal and non-fatal stroke (hazard ratio)	-	-	0.68 (0.46, 1.00)	-
Hospitalization for heart failure (hazard ratio)	-	-	1.03 (0.75, 1.40)	-
Aroda et al. [22]				
Patients with at least one gastrointestinal adverse event (SUSTAIN)	39.1%-41.9%	22.0%-24.8%	-	-
Serious or severe adverse events (SUSTAIN)	6%-9%	4%-7%	-	-
Discontinuation rate (SUSTAIN)	7.80%	3.00%	-	-
Discontinuation rate (PIONEER)	8.70%	4.20%	-	-

The improvement in exercise function was evident in the mean change in the six-minute walk distance, with semaglutide showing a superior performance compared to placebo (21.5 m vs. 1.2 m, respectively; estimated difference 20.3 m; 95% CI 8.6 to 32.1; P<0.001) (Table 4) [19]. The hierarchical composite endpoint analysis further underscored the efficacy of semaglutide, demonstrating a higher win ratio in favor of semaglutide over placebo (1.72; 95% CI 1.37 to 2.15; P<0.001) (Table 4) [19]. Additionally, the mean percentage change in CRP levels revealed a substantial anti-inflammatory effect of semaglutide (-43.5% vs. -7.3% with placebo; estimated treatment ratio 0.61; 95% CI 0.51 to 0.72; P<0.001), suggesting a potential mechanism beyond symptomatic relief and weight loss (Table 4) [19]. However, it is crucial to consider safety outcomes. Serious adverse events (AEs) were reported in 13.3% of the semaglutide group and 26.7% of the placebo group, highlighting a favorable safety profile for semaglutide (Table 4). In conclusion, the systematic review supports the efficacy of once weekly semaglutide (2.4 mg) in treating HFpEF in patients with obesity. Semaglutide demonstrated significant improvements in symptoms, exercise capacity, and weight loss compared to placebo, providing a promising therapeutic option for this challenging patient population.

Pérez-Belmonte et al. focused on the comprehensive assessment of once-weekly semaglutide (0.25 mg initiation, up to 1.00 mg) in 136 obese patients with type 2 diabetes and chronic HF [21]. The study explores the impact of semaglutide on various aspects, including glycemic control, HF health status, anthropometric characteristics, treatment de-intensification, laboratory variables, and safety. The baseline characteristics revealed that metformin was the most frequently used oral glucose-lowering drug (59.6%), followed by dipeptidyl peptidase-4 (DPP-4) inhibitors (39.7%) and sodium-glucose cotransporter-2 (SGLT-2) inhibitors (38.2%) [21]. Semaglutide, a non-semaglutide GLP-1 receptor agonist, was administered to 33.8% of patients. Basal insulin was used by 63.2% of patients, with an average dose of 39.0 units per day, and insulin combinations were used by 13.8% before starting semaglutide [21].

Over the 12-month study period, once-weekly semaglutide demonstrated significant clinical benefits. There was a substantial improvement in the Kansas City Cardiomyopathy Questionnaire (KCCQ) total symptom score, increasing from 59.0 to 79.9 points (p<0.01) (Table 4) [21]. Concurrently, there was a significant reduction in the proportion of patients with New York Heart Association (NYHA) functional class III (40.4% to 16.2%, p<0.01) and N-terminal pro-brain natriuretic peptide (NT-pro-BNP) levels (969.5 ± 653.5 to 577.4 ± 322.1 pg/mL, p<0.01) (Table 4) [21]. Glycemic control also improved, with a notable reduction in fasting blood glucose and hemoglobin A1c (HbA1c). The proportion of patients with HbA1c <7% increased from 16.2% to 64.5% at 12 months (p<0.001) (Table 4) [21]. Semaglutide therapy was associated with a significant reduction in body weight (12.7 kg) and body mass index (BMI) (7.1 kg/m²), leading to a decline in the proportion of patients with obesity (BMI ≥30) to 50.8%. This weight loss positively correlated with the improvement in the KCCQ total symptom score (r=0.612, p<0.01) (Table 4) [21].

The safety profile of semaglutide was generally favorable, with 24.2% of patients reporting adverse drug reactions, primarily gastrointestinal (GI) disorders (Table 4) [21]. Twelve patients discontinued semaglutide due to these AEs. Notably, there were six major adverse cardiovascular events (3P-MACEs) associated with two cardiovascular deaths. Emergency department visits and hospitalizations due to HF, as well as all-cause hospitalizations, decreased significantly 12 months after starting semaglutide. In conclusion, Pérez-Belmonte et al. showed that once-weekly use of semaglutide demonstrated positive outcomes in terms of HF health status, glycemic control, and anthropometric characteristics in obese patients with type 2 diabetes and chronic HF [21]. The therapy exhibited a favorable safety profile, emphasizing its potential as a comprehensive treatment option for this challenging patient population [21].

Husain et al. conducted a post hoc analysis on two trials (SUSTAIN and PIONEER) to assess MACEs in patients taking semaglutide, compared with placebo [20]. The trials included 6480 subjects, with 3239 randomized to semaglutide and 3241 to placebo. In the combined population of SUSTAIN 6 and PIONEER 6, the incidence rates of MACEs were 3.1 and 4.2 events per 100 subject-years with semaglutide and placebo, respectively (Table 4) [20]. The hazard ratio (HR) for MACE was 0.76 (95% CI 0.62, 0.92) in favor of semaglutide (Table 4) [20]. Individual components of MACE had HRs consistently <1.0, although the upper limit of the 95% CI for non-fatal stroke approached 1.0 [20]. Combined analysis of fatal and non-fatal myocardial infarction (MI) events showed an HR of 0.89 (95% CI 0.67, 1.18). For fatal and nonfatal stroke events, the HR was 0.68 (95% CI 0.46, 1.00) [20]. Incidence rates for hospitalization for HF were comparable between semaglutide and placebo (HR 1.03; 95% CI 0.75, 1.40) (Table 4) [20]. In the combined population, HRs for MACE were consistently <1.0 across CV risk subgroups, except for those with prior HF (HR 1.06; 95% CI 0.72, 1.57) [20]. 95% CIs spanned 1.0 for subgroups including CV risk factors only, prior MI or stroke, and prior HF. In the combined SUSTAIN and PIONEER glycemic efficacy trials, the incidence rates for MACE were 0.7 and 0.9 events per 100 subject-years with semaglutide and comparator, respectively [20]. A sensitivity analysis, excluding subjects on certain antidiabetic therapies, showed an HR of 0.77 (95% CI 0.47, 1.27) for MACE with semaglutide versus comparators. In summary, this study shows a favorable cardiovascular profile for semaglutide compared to placebo in subjects with T2DM, as demonstrated by a lower incidence of MACE and consistent HRs across various subgroups. The findings support the cardiovascular safety of semaglutide in this patient population [20]. 

Aroda et al. encompassed the SUSTAIN phase IIIa program (excluding SUSTAIN 6) and the PIONEER phase IIIa trials (excluding PIONEER 6) [22]. In SUSTAIN, 3150 patients (2712 patient-years of exposure (PYE)) received once-weekly subcutaneous semaglutide 0.5 or 1.0 mg, while 1657 patients (1467 PYE) received an active comparator or placebo [22]. In PIONEER, 4116 patients (4379 PYE) received once-daily oral semaglutide 3, 7, or 14 mg, and 2236 patients (2335 PYE) received a comparator [22]. The analysis of safety and tolerability outcomes revealed that the proportions of patients experiencing at least one AE were slightly higher for semaglutide compared to comparators in both the SUSTAIN and PIONEER phase IIIa pools [22].

The most prevalent AEs were GI, with 39.1%-41.9% of patients receiving semaglutide reporting them, including nausea (15.4%-19.1%), diarrhea (10.1%-12.6%), vomiting (6.6%-7.5%), and constipation (5.8%-6.5%) [22]. Comparatively, 22.0%-24.8% of patients receiving comparators reported GI AEs [22]. Serious or severe AEs occurred in approximately 6%-9% and 4%-7% of patients across semaglutide and comparator arms, respectively (Table 4) [22].

The proportions of fatal and serious AEs were similar between semaglutide and comparators, with no discernible causal relationship or pattern regarding the timing of deaths. Similar findings were observed in the placebo pools. GI events, notably nausea, tended to occur early during treatment, more prominently at higher doses and during dose escalation. However, these events diminished following continued use at each dose level. Exposure-response analyses indicated a relationship between dose and both nausea and vomiting. A higher proportion of patients receiving semaglutide discontinued the trial prematurely compared to those receiving comparators (7.8% vs. 3.0% in SUSTAIN and 8.7% vs. 4.2% in PIONEER for Semaglutide vs. comparators, respectively) (Table 4) [22]. GI AEs, including nausea, diarrhea, and vomiting, were the primary reasons for discontinuation. Discontinuation mainly occurred early in the treatment process and during dose escalation. 

Thus, it is observed that despite adverse effects, semaglutide is a potential drug for treating HF patients.

Discussion

The systematic review conducted here shows semaglutide as a promising and potential option in the comprehensive management of MACEs in T2DM. The qualitative results obtained from various included studies underline significant improvements in crucial clinical parameters such as in the KCCQ-CSS (p< 0.001), body weight (p< 0.001), six-minute walk distance (p< 0.001), and CRP levels (p< 0.001), demonstrating a promising role of semaglutide in the management of MACEs as well as HF with preserved ejection fraction (HFpEF). The literature has proposed various mechanisms to determine the role of GLP1 agonists on the cardiovascular system and decrease MACE [23]. Various factors, directly or indirectly, contribute to changes in the occurrence of MACE, as discussed ahead.

Heart Failure

The PIONEER 6 trial comparing the once-daily oral semaglutide with placebo showed a non-significant decreased risk of hospital admissions due to HF (HR 0.86 [0.48-1.55]; CI 95%) [24]. The SUSTAIN 6 trial, comparing Semaglutide 0.5mg and 1mg versus placebo doses of 0.5mg and 1mg, showed a non-significant increase in hospitalization rates for HF (HR 1.11 [0.77-1.61]; CI 95%; p=0.57) [17].

Hussain et al., 2019, in their meta-analysis, assessing the effect of cardiovascular outcomes trials (CVOTs) of different GLP-1 agonists, including the Semaglutide trials SUSTAIN 6 and PIONEER 6, reported a non-significant increase in hospitalization rates for HF with Semaglutide versus place (HR 1.03 [0.75-1.40]; 95% CI) [24].

Giugliano et al. in their meta-analysis, showed a significant decrease in HF incidence (HR 0.90 [0.83-0.98]; 95% CI; p= 0.023 [25]. Perez-Belmonte et al. reported significant improvements in the KCCQ-CSS (p< 0.001), a significant reduction in patients with New York Heart Association functional class 3 (NYHA-III) (p< 0.01) and pro-brain natriuretic peptide levels (p< 0.01) with once-weekly subcutaneous semaglutide [21]. Kosiborod et al. reported a significant change in the KCCQ-CSS (p< 0.001) [19].

The mean change in the six-minute walk distance was 21.5 m with subcutaneous semaglutide and 1.2 m with placebo (P< 0.001) [19].

Cardiovascular Outcomes

GLP 1 activity also reduces endothelial dysfunction as well as modulates angiogenesis [26,27]. GLP-1 agonist liraglutide has shown to increase ischemia-induced angiogenesis in murine models paving a step forward in the development of therapeutic angiogenesis for vascular and cardiovascular diseases [28,29].

The SUSTAIN 6 trial comparing the once-weekly subcutaneous semaglutide with placebo showed that semaglutide results in a significant (HR 0.74 [0.58-0.95]; 95%CI; p< 0.001 for noninferiority; p= 0.02 for superiority) decrease in the overall adverse cardiovascular outcomes (a composite of cardiovascular death, non-fatal myocardial infarction, or non-fatal stroke) as compared to placebo. This effect can be attributed to the significant (HR 0.61 [0.38-0.99]; 95% CI; p=0.04) decrease in the rate of nonfatal stroke and a nonsignificant (HR 0.74 [0.51-1.08]; 95% CI; P=0.12) decrease in the rate of non-fatal myocardial infarction, in patients given Semaglutide as compared to placebo, with no difference in deaths related to cardiovascular causes (HR 0.98 [0.65-1.48]; 95% CI; p= 0.92) [17]. The PIONEER 6 trial comparing the once-daily oral semaglutide with placebo, showed a significant noninferiority (HR 0.79 [0.57-1.11]; 95% CI; p< 0.001 for noninferiority; p= 0.17 for superiority) for primary outcomes (a composite of death from cardiovascular causes (including undetermined causes of death), nonfatal myocardial infarction, or nonfatal stroke) [24].

Husain et al., 2019, in their meta-analysis, assessing the effect of CVOTs of different GLP-1 agonists, including the semaglutide trials SUSTAIN 6 and PIONEER 6, reported a significant reduction in the incidence of non-fatal stroke (HR 0.65 [0.43-0.97]; 95% CI), non-significant reductions in fatal myocardial infarction (HR 0.78 [0.56-1.10]; 95% CI) and non-fatal myocardial infarction (HR 0.88 [0.66-1.18]; 95% CI) [24]. In the combined analysis of SUSTAIN 6 and PIONEER 6, the incidence rates of fatal and non-fatal MI events were 1.7 and 1.9 events per 100 subject-years with semaglutide and placebo, respectively (HR 0.89 [95% CI 0.67, 1.18]). For fatal and non-fatal stroke events, these were 0.8 and 1.2 events per 100 subject-years (HR 0.68 [95% CI 0.46, 1.00] [24].

Giugliano et al. included in their meta-analysis eight CVOTs with different GLP-1 agonists including two trials of semaglutide, and one trial each for lixisenatide, liraglutide, exenatide, albiglutide, dulaglutide, and efpeglenatide. The meta-analysis showed an overall 14% reduction (HR 0.86 [0.79-0.94]; 955 CI; P= 0.006) in MACE with a significant decrease in non-fatal stroke (HR 0.84 [0.76-0.94]; 95% CI; p= 0.007) and a nonsignificant decrease in non-fatal myocardial infarction (HR 0.91 [0.81-1.01]; 95% CI; p= 0.078) [25].

Aroda et al. in their study showed a decrease in the risk of MACEs, more with subcutaneous semaglutide than its oral form, which also met a non-inferiority criterion in terms of safety [22].

Effect on Obesity, Blood Pressure, and CRP Level

Obesity is associated with an increased risk of MACEs as it causes an inflammatory state, reduces nitric oxide, and provides a favorable environment for atherosclerotic activity [30]. A study by Ojeniran et al. has shown a 10-15% reduction in total body weight after Semaglutide exposure compared to placebo, thereby reducing MACE risk [31]. Kosiborod et al. reported a significant change in the Kansas City Cardiomyopathy Questionnaire clinical summary score (P< 0.001), and a significant decrease in body weight (P< 0.001) [19].

The GRADE trial assessed four mediations currently in use with metformin for the maintenance of type 2 diabetes, including insulin glargine, a long-acting insulin analog; liraglutide, a GLP-1 agonist; glimepiride, a sulfonylurea; and sitagliptin, a DPP-4 antagonist. The study found that Glargine and liraglutide were more effective than glimepiride and sitagliptin in achieving glycemic targets with the glycated hemoglobin level of 7.1% in both the glargine and liraglutide groups, 7.2% in the sitagliptin group and 7.3% in the glimepiride group after 4 years of treatment [32]. Meier et al. assessed both SUSTAIN trials for the subcutaneous formulation of Semaglutide and PIONEER trials for the oral formulation of semaglutide, for patient satisfaction through patient-reported outcomes in the form of Diabetes Treatment Satisfaction Questionnaire (DTSQ) for treatment satisfaction, and 36-item Short-Form Survey (SF-36) version 2 to assess physical function, pain, general health, mental health, emotional function, and social function, and found similar results of favoring the treatment when compared to placebo, and favoring oral formulation more than the subcutaneous one [33].

Considering high blood pressure as a major risk factor for developing MACEs, GLP 1 agonists have been shown to decrease both systolic and diastolic blood pressures. Drucker et al., in their study on exenatide, a GLP1 agonist with a similar mechanism of action to semaglutide, showed that the systolic blood pressure dropped by a mean of 4.7 mmHg (-6.9 - -2.6; 95% CI) and 3.4 mmHg (-5.5 - 1.3; 95% CI) for once a week and twice a day dose respectively, while the diastolic blood pressure decreased by 1.7 mmHg (-3.1 - -0.3; 95% CI) for once a week and twice a day dose [34,35].

Verma et al., in their three-step study, reported that Semaglutide 2.4 mg reduced CRP at week 68 versus placebo (estimated treatment difference −44% [-49 to −39; CI 95%] in Step 1, -39% [-46 to −30; CI 95%] in STEP 2, and -48% [-55 to −39; CI 95%] in STEP 3; all p < 0.05) [36].

Adverse Effects

Adverse GI disorders were reported in patients given Semaglutide with 41.9% effects in patients given subcutaneous Semaglutide and 39.1% in patients given Semaglutide through the oral route. Most of these adverse effects decreased with continued therapy [22]. 24.2% of patients given the drug had adverse GI effects [21]. Serious AEs were reported in 13.3% of participants in the Semaglutide group as compared to 26.7% of patients in the placebo group [19].

Our study has limitations, including a smaller number of articles and patients analyzed, as well as the inclusion of one observational study with three other articles. Nevertheless, these articles were pivotal and showed coherence in their findings, underscoring their importance for our manuscript.

## Conclusions

In conclusion, this systematic review underscores the promising efficacy of semaglutide in decreasing the incidence of HF, though it does not significantly affect the hospitalization burden associated with the condition. Objective improvements in HF management were observed through notable enhancements in the KCCQ-CSS, reductions in body weight, and increased six-minute walk distances. Furthermore, a comprehensive evaluation of clinical trials revealed significant improvements in cardiovascular events, including reductions in both fatal and non-fatal myocardial infarctions and strokes, alongside decreased CRP levels. Despite the occurrence of some adverse effects, semaglutide stands out as a potential therapeutic option for patients with HF. These findings highlight the importance of semaglutide in improving various cardiovascular parameters, suggesting its potential to contribute significantly to HF management. However, it is crucial to acknowledge the need for further research and long-term monitoring to better understand the full scope of semaglutide and other GLP1 agonists' impact in this context. Continued investigation will help refine our knowledge of its benefits and risks, paving the way for more effective and comprehensive cardiovascular care strategies. Thus, ongoing research is essential to ensure that healthcare providers can deliver optimal treatment to patients with HF, ultimately enhancing patient outcomes and quality of life.
